# Feelings of Guilt and Remorse After Alcohol Consumption Among People Who Drink at Increasing and Higher‐Risk Levels: A Population Study in England

**DOI:** 10.1111/dar.70076

**Published:** 2025-11-27

**Authors:** Sharon Cox, Melissa Oldham, Harry Tattan‐Birch, Sally Marlow, Deborah Robson, Claire Garnett, Sarah Jackson

**Affiliations:** ^1^ Department of Behavioural Science and Health University College London London UK; ^2^ Behavioural Research UK Edinburgh UK; ^3^ Institute of Psychiatry, Psychology and Neuroscience, King's College London London UK; ^4^ School of Psychological Science, University of Bristol Bristol UK

**Keywords:** alcohol, AUDIT, drinking behaviours, guilt, remorse

## Abstract

**Introduction:**

Feelings of guilt and remorse after drinking alcohol may act as barriers to seeking support. This study aimed to estimate the prevalence and frequency of such feelings among adults in England who drink at increasing and higher‐risk levels, and differences by socio‐demographic and drinking subgroups.

**Methods:**

We analysed data from 40,708 adults (≥ 18 years) who drink at increasing and higher‐risk levels (AUDIT‐C score ≥ 5) from a monthly cross‐sectional survey in England from 2014 to 2022. Feelings of guilt and remorse after drinking in the past 6 months were assessed using the AUDIT. Logistic regression models were used to examine associations with socio‐demographic characteristics, alcohol consumption and harm to oneself or others as a result of drinking.

**Results:**

Overall, 13.3% (95% CI 12.9%–13.6%) reported experiencing feelings of guilt or remorse after drinking in the past 6 months. Among those who reported such feelings, 95.3% (95% CI 91.4%–94.9%) experienced them less than once a month. The prevalence of guilt and remorse increased non‐linearly with higher AUDIT‐C scores from 9.3% (95% CI 8.8%–9.9%) among those drinking at the lightest levels within the increasing/higher‐risk range [AUDIT‐C = 5] to 20.9% (95% CI 17.2%–24.8%) among the heaviest [AUDIT‐C = 12]. After adjusting for alcohol consumption and alcohol‐related injury, these feelings were more common among women (aOR 1.38; 95% CI 1.31–1.46) and people from more advantaged social grades (aOR 1.27; 95% CI 1.20–1.36), and much less common among older than younger adults (e.g., ≥ 65 vs. 16–24 years: aOR 0.23; 95% CI 0.20–0.26).

**Discussion and Conclusions:**

In England, around one in eight adults who drink at increasing and higher‐risk levels report experiencing guilt or remorse after drinking. These feelings are more common in women, younger adults and those of a more advantaged social grade.

**Summary**

- One in eight increasing and higher‐risk drinkers reported guilt or remorse after drinking.
- Feelings of guilt were more common in women, younger and advantaged groups.
- Guilt increased with higher alcohol consumption, regardless of demographics.
- Future work should explore drinking patterns and cultural influences on guilt.

## 1 Introduction

Globally, alcohol consumption is a leading risk factor for poor health, disability and premature death [1]. Despite its harms, alcohol has a strong sociocultural position within British life [2]. In England, one in three adults drink at ‘increasing and higher risk’ levels, which the Office for Health Improvement and Disparities defines as placing people at an especially elevated risk of alcohol‐related health harm [3, 4]. The COVID‐19 pandemic led to a greater number of people drinking at increasing and higher‐risk levels, for example, an AUDIT‐C score of ≥ 5 [5], with some signs that this level of drinking has been maintained since this period [3]. In 2021, the UK recorded an all‐time high number of alcohol‐attributable deaths [6]. Helping people to reduce their alcohol consumption and exploring potential barriers to reduction is a public health priority.

Although alcohol is a commonly used substance, people may feel guilt or remorse about their drinking due to both the harm it causes to themselves and others and the stigma that can surround heavier drinking. Guilt is a process of reasoning that casts a person's behaviour or thoughts as morally wrong, bad or inappropriate (e.g., I should not want to drink, I am bad for drinking) [7]. Remorse is the feeling of guilt and regret for a past action [8] and a wish that the behaviour or event had not happened or that there was another outcome (e.g., I wish I hadn't drunk so much, I wish I hadn't acted in that way). People may feel guilt and remorse because they drink more than they think they should, because their drinking harms them or because they believe their drinking is harming others or interfering with other responsibilities, such as work or education. In some cases, people may drink at risky levels to avoid feeling the very feelings of guilt and remorse that drinking generates, but this may be a vicious cycle, as continual drinking may create and maintain the feelings one is trying to forget. Understanding how guilt and remorse about drinking operate across different subgroups of the population is important because clinical studies have shown these feelings can preclude the likelihood of someone seeking help for their drinking [9, 10, 11].

It is plausible that the more alcohol someone consumes, the more likely they are to feel guilt or remorse after drinking. In England, average alcohol consumption increases with age, with adults aged ≥ 45 years being among the heaviest and those aged 16–24 consuming the lowest levels [12]. While there is little evidence on how guilt and remorse after drinking vary across the life course or between ages, some evidence suggests that younger adults are less likely to seek support for their drinking compared with older adults (≥ 50 years), often reporting a belief that they can manage the issue themselves [12]. There may be differences in the types of support needed by age group, and if there are widely varied levels of guilt and remorse, this may mean the approach needs to be somewhat tailored.

There is substantial evidence that alcohol consumption varies by gender and sex. Surveys from England have shown that men tend to consume both more and more frequently than women [12]. In 2022, 30% of men reported exceeding the weekly alcohol limit (which is defined as 14 units for both men and women), versus 15% of women and 13% of men compared with 8% of women drank alcohol on at least 5 days in the previous week [12]. However, the evidence on how guilt and remorse manifest in men and women is less clear. Some clinical studies show no gender differences [13], whereas others suggest women experience more guilt and remorse [14]. Historically, women who drink and especially those who have sought clinical support for their alcohol use have faced social stigma and scrutiny, for example, perceived as uncontrolled or as a transgression of their femininity [10, 11, 15, 16]. For those women with children, drinking can be seen as unmotherly and uncaring, leading to judgement from others [17] or fear that they may be judged [11]. Such social stigma may mean women who could benefit from help with their drinking do not seek it or hide their drinking, which may, in turn, further increase feelings of guilt and remorse around their drinking [11, 16]. There is less literature on how guilt and remorse affect men and their drinking. Some men feel pressure to drink heavily [18], in ways which relate to their social identity and cultural norms [18, 19]. Heavy drinking may be seen as normal or given tacit approval within social networks [20]. Despite this, some men do feel guilty as a result of their drinking, with some studies showing men report the same amount of guilt as women, at least in clinical samples [13]. However, men are generally less likely to seek health advice, and any barriers to improving their health must be understood [21].

To date, evidence on these feelings among people who drink has come from clinical samples, and there is less evidence from national surveys. This is an important gap in the literature, because while many people drink at levels that could cause harm and negative affect, many people do not seek support for their drinking, and evidence to date may not be widely representative. In this study, using cross‐sectional data from a nationally representative survey in England, we explored the prevalence of past 6‐month self‐reported feelings of guilt and remorse after alcohol consumption (as measured by the AUDIT), associated socio‐demographic and drinking characteristics, and differences by gender among adults who drink at increasing and higher‐risk levels. Specifically, we addressed the following research questions:

RQ1: What proportion of adults who drink at increasing and higher‐risk levels report experiencing feelings of guilt and remorse after drinking in the past 6 months?

RQ2: Among those who report feelings of guilt and remorse after drinking in the past 6 months, how frequently are these feelings experienced?

RQ3: To what extent do feelings of guilt and remorse after drinking differ by socio‐demographic characteristics (age, gender, social grade), and alcohol‐related injury to oneself or others?

RQ4: To what extent are differences in the proportion reporting feelings of guilt and remorse after drinking between socio‐demographic groups explained by differences in alcohol consumption and alcohol‐related injury?

## 2 Methods

### 2.1 Pre‐Registration and Ethics

The protocol for this study was pre‐registered on the Open Science Framework https://osf.io/c93te. We had initially intended to estimate prevalence across all adults who drink. However, because questions on guilt and remorse were only asked of participants who drank at increasing or higher‐risk levels (AUDIT‐C score ≥ 5), this would have meant imputing this variable as 0 (i.e., no feelings of guilt or remorse) for those scoring below 5 on the AUDIT‐C, which may underestimate the true prevalence. We therefore made the decision to limit the analyses to participants who drank at increasing or higher‐risk levels. In addition to the pre‐registered analyses, we also present the proportion of those feeling guilt and remorse in the past 6 months, overall and by subgroups, as a function of alcohol consumption. Following review, we also include the survey year as a covariate.

Ethical approval was provided by the UCL Research Ethics Committee (0498/001). Participants provided informed consent to take part in the study, and all methods were carried out per relevant regulations. The data are not collected by UCL (collected by a third party, Ipsos MORI) and are anonymised when received.

### 2.2 Sample and Recruitment

Data were drawn from the Alcohol Toolkit Study [22], an ongoing monthly cross‐sectional survey of a nationally representative sample of adults in England. The study used a hybrid of random probability and simple quota sampling to select a new sample of approximately 1700 adults each month. Full details of the sampling procedure are provided elsewhere [22].

Data were collected monthly through face‐to‐face computer‐assisted interviews up to February 2020. However, social distancing restrictions under the COVID‐19 pandemic meant that no data were collected in March 2020, and data from April 2020 onwards were collected via telephone. The telephone‐based data collection relied upon the same combination of random location and quota sampling, and weighting approach as the face‐to‐face interviews, and comparisons of the two data collection modalities generally indicated good comparability [23].

For the present study, we used data collected from respondents to the survey in the period from March 2014 (the first wave of the Alcohol Toolkit Study) to March 2022 (the most recent wave with the relevant data available). We restricted the sample to those aged ≥ 18 years (the legal age of sale of alcohol in England) who reported drinking at increasing and higher‐risk levels (AUDIT‐C ≥ 5).

### 2.3 Measures

Level of alcohol consumption was assessed with the AUDIT‐C (AUDIT questions 1–3). Scores ranged from 0 to 12, with higher scores indicating greater consumption. We restricted the sample to those with a score of ≥ 5. The three questions asked were:

1. How often did you have a drink containing alcohol in the past 6 months? Answer options were: Never, monthly or less, 2 to 4 times per month, 2 to 3 times per week, 4 or more times per week.
2. On days in the past 6 months when you drank alcohol, how many drinks did you typically drink? Answer options were: 0, 1 or 2, 3 or 4, 5 or 6, 7–9, 10 or more.
3. How often did you have 6 or more (for men) or 4 or more (for women and everyone 65 and older) drinks on an occasion in the past 6 months? Answer options were: Never, less than monthly, monthly, weekly, daily or almost daily.

Guilt and remorse were taken from the full Alcohol Use Disorders Identification Test (AUDIT). The AUDIT is a 10‐item screening tool for the identification of alcohol‐related harms and potential disorders. Only people who scored ≥ 5 on the AUDIT‐C (the first three questions of the AUDIT) were asked the remaining AUDIT questions. Question 7 assessed feelings of guilt and remorse asked participants: ‘How often during the last 6 months have you had a feeling of guilt and remorse after drinking?’ Participants selected one response: (i) daily/almost daily; (ii) weekly; (iii) monthly; (iv) less than monthly; (v) never; (vi) don't know; and (vii) refused. ‘Don't know’ and ‘refused’ were treated as missing. We presented data as any vs. no guilt (i.e., responses i–iv vs. v) for RQs 1, 3 and 4, and as each separate response option for RQ2.

Injury to oneself or others from drinking was derived from participants' responses to question 9 from the full AUDIT. Participants responded to the question: ‘Have you or somebody else ever been injured as a result of your drinking?’. Respondents answered yes, yes, but not in the last 6 months, or no. We categorised this as yes/no.

Gender was self‐reported as male, female or in another way. Those who identified in another way were included in descriptive analyses but removed from analyses by gender owing to small numbers.

Age was categorised as 18–24, 25–34, 35–44, 45–54, 55–64 or 65+ years.

Social grade was categorised according to the National Readership Survey [24] classification as AB (higher and intermediate managerial, administrative, and professional); C1 (supervisory, clerical, and junior managerial, administrative and professional); C2 (skilled manual workers); D (semi‐skilled and unskilled manual workers); or E (state pensioners, casual and lowest grade workers, unemployed with state benefits only). We categorised and reported this as ABC1 (more advantaged) and C2DE (less advantaged).

The survey year was categorised for each year from 2014 to 2022.

### 2.4 Statistical Analysis

The analyses were conducted in RStudio using R version 4.4.2. Descriptive data are presented on the socio‐demographic characteristics and alcohol‐related variables for adults who drank at increasing and higher‐risk levels. These are reported as unweighted for sample sizes, and weighted percentages alongside their 95% confidence intervals (CI) for categorical variables, and as means and standard deviations (SD) for continuous ones. Weighting was applied using a rim (marginal) weighting, or raking, procedure. This method iteratively adjusts respondent weights so that the weighted distributions of key socio‐demographic variables match known population benchmarks; further details are provided elsewhere [22].

For all logistic regression models, odds ratios (unadjusted and adjusted, where appropriate) were reported alongside their associated 95% CIs. Analyses were run on complete cases only (for clarity, the proportion of those excluded is reported).

RQs 1 & 3: The proportion (% and 95% CI) who reported experiencing feelings of guilt and remorse after drinking in the past 6 months was estimated; overall and by socio‐demographic characteristics (age, gender, social grade), level of alcohol consumption, and alcohol‐related injury to oneself or others.

RQ2: Among those who reported experiencing guilt and remorse after drinking, the frequency with which they had these feelings (less than monthly, monthly, weekly, daily/almost daily) was reported as proportions (% and 95% CI).

RQ4: Using logistic regression models, associations of feeling guilt and remorse after drinking (dependent variable) with socio‐demographic characteristics (age, gender, social grade; independent variables) and survey year (2014–2022) were analysed, fully and without adjustment.

## 3 Results

During the study period, 158,603 people responded to the survey, with 148,557 complete cases (2.1% missing cases (1.1% for AUDIT‐C, 2.0% feelings of guilt and remorse, 0.5% alcohol‐related injury)). The majority of people, 72.1% (*n* = 107,073, 95% CI 71.6%–72.3%), drank alcohol (AUDIT‐C ≥ 1), and 27.4% (*n* = 40,708, 95% CI 27.2–27.6) of the total sample drank at increasing and higher‐risk levels.

The weighted prevalence of experiencing guilt and remorse in the past 6 months was 13.3% (95% CI 12.9%–13.6%) among those who drink at increasing or higher‐risk levels. Among those who felt guilt, the majority felt this less than monthly (95.3%; 95% CI 91.4%–94.9%).

The weighted sample characteristics of the sample of people who drink at increasing and higher‐risk levels and who report feeling guilt and remorse in the past 6 months are presented in Table 1.

**TABLE 1:** Socio‐demographic characteristics of adults who drink at increasing and higher‐risk levels who report feeling any guilt and remorse over the past 6 months.

	Adults who drink at increasing and higher‐risk levels *N = 40,708	Adults who drink at increasing and higher‐risk levels who do not report feeling any guilt and remorse *N = 35,431	Adults who drink at increasing and higher‐risk levels who report feeling any guilt and remorse *N = 5277
Characteristic	% [95% CI]	
% [95% CI]
Age, years			
18–24	15.4 [15–16.1]	14.0 [13.3–15.7]	25.6 [24.1–27.6]
25–34	16.9 [16.3 17.2]	11.7 [10.5–13.0]	21.7 [20.9–23.9]
35–44	17.4 [17.1–18]	15.6 [14.5–16.7]	20.0 [19.3–21.2]
45–54	20.7 [20.2, 21.4]	19.1 [18.1–20.1]	17.7 [17.0–19.5]
55–64	15.7 [15.5–16.3)	14.9 [13.9–15.8]	9.6 [8.8–10.1]
≥ 65	14.0 [14–14.2]	13.5 [12.8–14.0]	5.5 [4.9–6.1]
Gender			
Men	64.2 [61.3–64.8]	65.9 [64.7–67.0]	57.8 [56.8–58.1]
Women	35.8 [35.2–36.1]	34.1 [33.0–35.3]	42.2 [41.9–44.4]
Social grade			
ABC1	62.7 [61.2–63.2]	60.5 [59.2–61.8]	67.1 [65.8–68.1]
C2DE	36.6 [36.3–37]	37.1 [36.1–38.1]	32.9 [31.3–34.9]
AUDIT‐C category^a^			
Increasing risk: 5–7	81.7 [81–82.3]	82.9 [81.9–83.9]	74.6 [73.5–77.8]
Higher risk: 8–12	18.3 [18–19.1]	17.1 [16.1–18.1]	26.4 [23.9–27.1]
Survey year			
2014	11.0 [10–11.9]	10.7 [9.9–11.6]	8.6 [7.9–9.2]
2015	11.3 [10.4–12.2]	10.8 [10.0–11.6]	9.2 [8.5–9.8]
2016	12.5 [11.5–13.4]	12.2 [11.3–13.1]	9.7 [9.1–10.4]
2017	12.5 [11.6–13.4]	12.2 [11.3–13.2]	9.5 [8.9–10.2]
2018	12.2 [11.3–13.1]	11.9 [11.0–12.8]	9.1 [8.5–9.8]
2019	13.4 [12.4–14.4]	13.2 [12.3–14.2]	10.3 [9.6–10.9]
2020	14.2 [13.2–15.1]	14.1 [13.2–15.1]	10.8 [10.1–11.4]
2021	15.1 [14.2–16.1]	15.0 [14.1–16.0]	11.6 [10.9–12.2]
2022	15.9 [14–17.9]	15.7 [14.0–17.4]	12.9 [11.5–14.3]
Guilt frequency			
Never	86.7 [86.4–87.1]		
Less than monthly			93.5. [91.4–94.9]
Monthly			2.20 [1.91–2.33]
Weekly			0.50 [0.50–0.51]
Daily or almost daily			3.80 [3.41–4.10]
Alcohol‐related injury			
No	90.5 [90.4–90.6]	92.1 [91.2–93.0]	71.7 [70.4–73.3]
Yes	9.5 [9.1–9.6]	7.9 [7.1–8.7]	28.3 [27.5–30.3]

*Note:* Percentages and 95% confidence interval account for survey weights (column percentages are presented). *Sample size is shown for unweighted data.

Abbreviations: AUDIT, Alcohol Use Disorders Identification Test; CI, confidence interval.

^a^ Participants were only asked about their guilt and remorse after drinking if they had AUDIT‐C scores of at least 5.

Table 2 presents the unadjusted and adjusted associations, which show that even when adjusting for alcohol consumption and alcohol‐related injury, the odds of experiencing feeling guilt or remorse in the last 6 months were higher among those who were female (aOR 1.38; 95% CI 1.31–1.46) and from more advantaged social grades (aOR 1.27; 95% CI 1.20–1.36). Older adults (≥ 65 years) were the least likely to feel any guilt and remorse and less likely than younger adults (those between the ages of 18–24 years; aOR 0.23; 95% CI 0.20–0.26).

**TABLE 2:** Logistic regression (unadjusted and adjusted) of feeling guilt and remorse after drinking with socio‐demographic characteristics among people who drink at increasing and higher‐risk levels (*N* = 40,708).

	Feeling guilt and remorse after drinking in the past 6 months		
	% [95% CI]^a^	Unadjusted OR [95% CI]	Adjusted^b^ OR [95% CI]
Age, years			
18–24	22.3 [21.3–23.3]	Ref	Ref
25–34	17.3 [16.3–18.3]	0.55 [0.50–0.59]	0.74 [0.68–0.80]
35–44	15.5 [14.6–16.5]	0.52 [0.48–0.57]	0.62 [0.57–0.68]
45–54	11.7 [11.0–12.4]	0.45 [0.42–0.49]	0.47 [0.43–0.51]
55–64	8.2 [7.60–8.90]	0.29 [0.26–0.32]	0.35 [0.31–0.39]
≥ 65	4.9 [4.42–5.43]	0.10 [0.092–0.11]	0.23 [0.20–0.26]
Gender			
Men	11.4 [11.0–11.8]	Ref	Ref
Women	15.8 [15.2–16.4]	1.22 [1.21–1.24]	1.38 [1.31–1.46]
Social grade			
C2DE (less advantaged)	11.3 [10.7–11.8]	Ref	Ref
ABC1 (more advantaged)	13.8 [13.4–14.2]	1.82 [1.72–1.94]	1.27 [1.20–1.36]
Survey year			
2014	10.8 [10.2–11.3]	Ref	Ref
2015	10.1 [10–10.2]	1.08 [0.96–1.21]	1.11 [0.98–1.25]
2016	12.5 [12–13]	1.15 [1.03–1.29]	1.16 [1.03–1.31]
2017	12.9 [11.9–13.1]	1.13 [1.00–1.26]	1.15 [1.03–1.30]
2018	11.8 [11.3–12.7]	1.07 [0.96–1.20]	1.12 [0.99–1.26]
2019	13 [12.8–13.6]	1.22 [1.09–1.36]	1.29 [1.14–1.45]
2020	14.9. [13.4–15]	1.29 [1.15–1.44]	1.34 [1.19–1.50]
2021	15.6 [15.2–15.8]	1.39 [1.25–1.55]	1.41 [1.26–1.58]
2022	15.4 [15–16.4]	1.58 [1.35–1.84]	1.64 [1.40–1.92]
Alcohol consumption^c^			
AUDIT‐C (per unit)	1.26 [1.23–1.34]	1.31 [1.30–1.34]	1.23 [1.21–1.25]
Alcohol‐related injury			
No	71.7 [70.4–73.3]	Ref	Ref
Yes	28.3 [27.5–30.3]	2.55 [2.35–2.61]	2.30 [2.15–2.45]

Abbreviations: AUDIT, Alcohol Use Disorders Identification Test; CI, confidence interval; OR, odds ratio.

^a^ % and CI are adjusted prevalence estimates (shown as row percentages).

^b^ Adjusted for alcohol consumption (AUDIT‐C), alcohol‐related harm to oneself or others, age, gender and social grade.

^c^ Odds ratio f^2^or alcohol consumption shows increase in odds of feeling guilt and remorse for every one‐unit increase in AUDIT‐C.

Adjusted logistic regression results showed that the odds of feeling guilt and remorse after drinking increased by 23% (aOR = 1.23, 95% CI 1.21–1.25) for every one‐unit increase in AUDIT‐C (Table 2). This pattern of increasing prevalence with higher AUDIT‐C scores was consistent across demographic subgroups, including gender, social grade and age (see Supporting Information).

**FIGURE 1:**
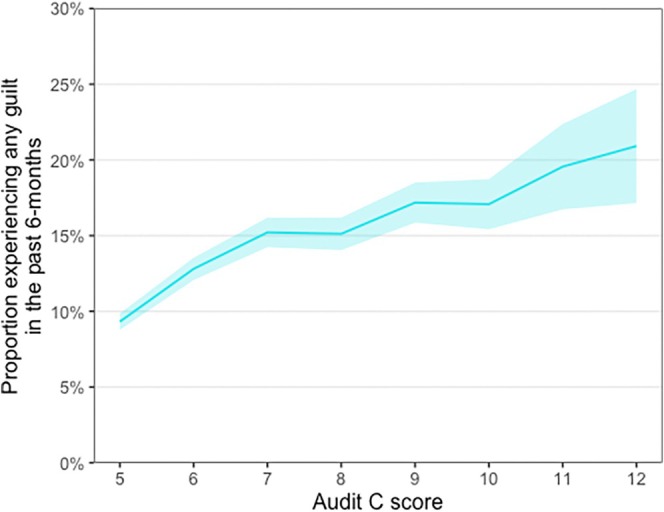
Proportion of adults who report experiencing guilt and remorse after drinking in the past 6 months by AUDIT‐C score. Line represents prevalence and shaded area the 95% confidence intervals. AUDIT, Alcohol use disorders identification test.

Unplanned exploratory analyses, presented in Figure 1, showed the prevalence of guilt and remorse increased with higher alcohol consumption, from 9.3% (95% CI 8.8%–9.9%) among those drinking at the lightest levels within the increasing/higher‐risk range [AUDIT‐C = 5] to 20.9% (95% CI 17.2%–24.8%) among the heaviest [AUDIT‐C = 12] (Figure 1).

## 4 Discussion

This study explored feelings of guilt and remorse after drinking among adults in England who drink at increasing and higher‐risk levels. The study found that just over one in eight individuals in this group reported feelings of guilt and remorse in the past 6 months, although the majority experienced them less than monthly. The prevalence of guilt and remorse increased with higher levels of alcohol consumption and differed across demographic subgroups; it was higher among women and in more advantaged social grades, and much higher in younger adults.

While overall feelings of guilt and remorse were more prevalent in those with higher alcohol consumption (as indexed by AUDIT‐C score), there were notable differences between men and women. Despite men consuming more alcohol, as reflected in higher AUDIT‐C scores, women reported higher levels of guilt and remorse. Previous research has highlighted the role of social stigma and gender norms in shaping feelings of guilt and remorse, particularly among women [10, 11, 15]. The observed differences here are consistent with the literature from clinical samples and settings, as well as other surveys showing that women do feel more guilt and remorse (or regret [25]). While our data do not tell us why, past literature has linked this to women's remorse around vulnerability [10, 16]. While previous studies have often framed women's guilt in terms of physical vulnerability when drinking, guilt is also closely tied to broader social expectations of femininity and motherhood. For mothers, drinking can be seen as incompatible with caring roles, leading to fears of being judged as ‘unmotherly’ or irresponsible [17]. Among younger women in higher education, guilt is reported in relation to binge episodes and the difficulty of balancing social drinking with academic or personal responsibilities [26]. These pressures suggest that women's guilt is shaped not only by individual consumption but also by cultural and relational contexts, which may explain why women in our sample reported higher levels of guilt despite consuming less on average than men. As women's drinking has risen in recent years [3, 12], and a higher proportion of women are drinking at increasing and higher‐risk levels [12], it is important to understand the potentially complex feelings women experience around their alcohol use, especially feelings that may act as a barrier or catalyst for change.

Younger adults, particularly those aged 18–24 years, were more likely to report feelings of guilt and remorse after drinking compared with older adults. This could be due to different social expectations and pressures around alcohol use faced by younger individuals [25]. Over the last 30 years, there have been large declines in alcohol consumption in younger age groups [27], accompanied by changing discourses about alcohol's role in people's lives [28]. Alcohol is increasingly seen, by both people who drink and those who do not, as a coping mechanism [25, 28], and drinking to cope can be interpreted as a sign of problematic use, which may in turn elicit greater feelings of guilt. There also may be cohort effects, whereby older adults have developed a different relationship with alcohol due to social and cultural differences developed when they were younger and since, and/or different ways of conceptualising the role that alcohol plays within their lives as they grow older. Some evidence also suggests that older adults may have developed more robust ways of dealing with negative emotions, irrespective of alcohol use, and these coping mechanisms can help people to become less self‐critical [29].

People from more advantaged social grades (ABC1) also reported higher levels of guilt and remorse after drinking compared with those from less advantaged grades (C2DE). This might reflect differences in social norms and expectations around drinking behaviour, although it warrants further exploration.

This study shows that feelings of guilt and remorse are more prevalent in certain subgroups, suggesting that tailored interventions which account for people's socio‐demographic backgrounds may be more effective in supporting help‐seeking. Evidence from other fields indicates that incorporating components specifically designed to address guilt can enhance the impact of existing interventions [30]. It is also important to consider the role of shame, which we do not include as a measure here, as our measures are from the AUDIT, which does not assess shame. However, some theories which differentiate these feelings suggest guilt may motivate constructive change, whereas shame is more often associated with concealment or withdrawal [31]. Our measure of guilt and remorse may capture elements of both; the implications for behaviour could differ. Recent debates on ‘justified disapproval’ in alcohol policy highlight this: feelings of guilt may be seen as useful levers for change, but interventions that inadvertently elicit shame risk reinforcing stigma and disengagement [32]. There is also a broader argument, beyond drinking but relevant for a wide range of behaviour change interventions, that stigma and the arising psychological feelings it creates, are ever justifiable regardless of the outcome.

For younger adults and women in particular, interventions may need to confront the social pressures and stigma associated with drinking. One promising approach for individuals experiencing guilt or shame is offering anonymous support. For example, in a qualitative evaluation of the ‘Drink Less’ app, users reported reluctance to disclose their alcohol consumption to health professionals—whether in person or through data‐linked systems—due to fears of judgement or consequences [33]. Anonymous or digital interventions, for example, may provide safer spaces for individuals to process feelings of guilt or remorse without fear of judgement, thereby reducing barriers to seeking support [29].

There are several strengths to this study. First, data were drawn from a nationally representative study and are unique in measuring guilt and remorse within population surveys. There are also some limitations. First, although our measure of guilt and remorse comes from the AUDIT, which is a well‐used tool, we measure it through a single item, and, more still, throughout the literature, differing definitions of guilt have been used [34] making findings harder to compare. Second, due to small sample sizes within the number of people who report frequent (e.g., weekly) guilt, we cannot meaningfully provide gender, social grade or differences in the frequency of these feelings. Third, our data are confined to people who drink at increasing and higher‐risk levels, a sub‐sample of adults who may not represent the total population of people who drink; therefore, our results may be an underestimation of the population levels of these feelings. Fourth, a large majority of people reported feeling guilt or remorse less than monthly, and while this may be true, this is a sensitive question, and answers may vary under different (more familiar) contexts. There may also be an element of recall bias. Fifth, we know interactions such as mental well‐being and illness, as well as other demographic factors such as ethnicity and religion, can play a role in feelings of guilt and remorse [25] (in both directions), as well as alcohol use. We acknowledge that larger cross‐sectional surveys such as ours are not always the most appropriate design for exploring differences by ethnicity or other minority characteristics. In our study, the relatively small sample sizes within some groups meant that any analyses would have produced imprecise or potentially misleading estimates; therefore, we did not include these variables. Nevertheless, we recognise these are important issues, and future research, including studies with larger targeted samples or qualitative approaches, could usefully explore how ethnicity and cultural context shape experiences of guilt and remorse. Additionally, we did not separately examine the frequency and quantity components of drinking patterns (e.g., regular moderate use versus episodic heavy drinking), which may differentially relate to feelings of guilt and remorse. Future research could explore how these specific drinking behaviours shape guilt and remorse across different age groups. Last, our study is cross‐sectional, and while we have posited several theories as to why some people experience more guilt and remorse than others, our survey design cannot establish causality.

## 5 Conclusion

Just over one in eight adults in England who drink at increasing or higher‐risk levels reported guilt or remorse after drinking. These feelings were more prevalent among those with greater alcohol consumption, women, younger adults and those from more advantaged social grades. While we do not know if addressing guilt and remorse does improve interventions, the findings highlight the need for nuanced public health strategies which account for potential barriers that these feelings may trigger.

## Author Contributions

S.C. and S.J. conceptualised the study. All authors contributed to the methodology. S.C. led the data analysis. S.J. acted as study oversight. S.C. led the writing of the manuscript and all authors contributed to the revising of the manuscript.

## Conflicts of Interest

S.C., D.R., H.T.‐B., S.M. and S.J. have no conflicts of interest to declare. M.O. has received funding from Alcohol Change UK (ACUK) for a separate ongoing research project on alcohol‐free and low‐alcohol drinks that began in January 2025. C.G. worked on a project funded by Alcohol Change UK (ACUK) that ran from September 2021 to March 2023, with a no‐cost extension running until March 2025. Since beginning those projects, the authors have become aware that ACUK received < 0.6% of its funds in 2024–5 from Lucky Saint, an organisation that produces and sells non‐alcoholic drinks, and owns a pub that sells standard alcoholic drinks. In March 2025, Lucky Saint became an associate member of The Portman Group, a self‐regulatory organisation that is fully funded and controlled by the alcohol industry. ACUK has a strict policy of not accepting any funds from, nor being subject to any influence whatsoever from, the alcohol industry, including through its investment portfolio. ACUK has stated that it remains in full compliance with its policy.

## Supporting information


**Data S1:** dar70076‐sup‐0001‐Supinfo.docx.

## Data Availability

The data that support the findings of this study are available from the corresponding author upon reasonable request.
