# Supplementary material for: Feelings of Guilt and Remorse After Alcohol Consumption Among People Who Drink at Increasing and Higher‐Risk Levels: A Population Study in England
Source: Drug Alcohol Rev. 2025 Nov 27;45(1):e70076. doi: 10.1111/dar.70076 (PMC12660003; doi:10.1111/dar.70076)
Supplement: Supplementary file 1 — Data S1: dar70076‐sup‐0001‐Supinfo.docx. [file DAR-45-0-s001.docx]

**Supporting Information: Proportion of those who report experiencing any guilt over the past 6-months by AUDIT-C score. Line represents prevalence and shaded area the 95% confidence interval.** **AUDIT, Alcohol Use Disorders Identification Test.**


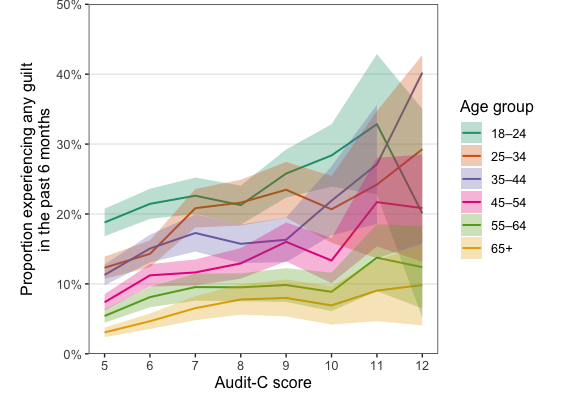


**
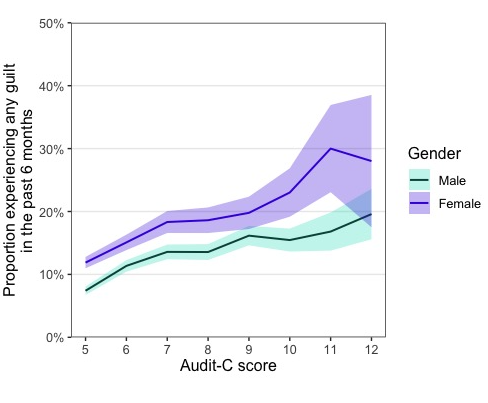

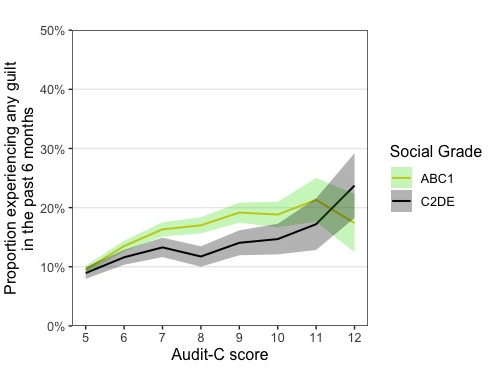
**

The plotted sub-sample data show non-linear increases in the proportion of those feeling guilt and remorse about their drinking as a function of AUDIT-C, higher among women than men. It was also higher among those of more advantaged social grade, and those between the ages of 18-24 years old, though less certain for those between the ages 25-34.
